# Sex Hormone-Binding Globulin (SHBG) Maintains Proper Equine Adipose-Derived Stromal Cells (ASCs)’ Metabolic Functions and Negatively Regulates their Basal Adipogenic Potential

**DOI:** 10.1007/s12015-023-10580-8

**Published:** 2023-07-04

**Authors:** Lynda Bourebaba, Magdalena Zyzak, Mateusz Sikora, Anna Serwotka-Suszczak, Malwina Mularczyk, Mohamad Al Naem, Krzysztof Marycz

**Affiliations:** 1 Department of Experimental Biology, Faculty of Biology and Animal Science, Wrocław University of Environmental and Life Sciences, Norwida 27B, 50-375 Wrocław, Poland; 2 Faculty of Veterinary Medicine, Equine Clinic - Equine Surgery, Justus-Liebig-University, 35392 Gießen, Germany; 3 Department of Veterinary Medicine and Epidemiology, Veterinary Institute for Regenerative Cures, School of Veterinary Medicine, University of California, Davis, CA USA

**Keywords:** SHBG, ASCs, Knockdown, Apoptosis, Antiadipogenic, Mitochondrial dynamics

## Abstract

**Background:**

Sex hormone binding globulin (SHBG) deteriorated expression has been recently strongly correlated to increased level of circulating pro-inflammatory cytokines and insulin resistance, which are typical manifestations of equine metabolic syndrome (EMS). Despite previous reports demonstrated the potential therapeutic application of SHBG for liver-related dysfunctions, whether SHBG might modulate equine adipose-derived stem/stromal cells (EqASCs) metabolic machinery remains unknown. Therefore, we evaluated for the first time the impact of SHBG protein on metabolic changes in ASCs isolated from healthy horses.

**Methods:**

Beforehand, SHBG protein expression has been experimentally lowered using a predesigned siRNA in EqASCs to verify its metabolic implications and potential therapeutic value. Then, apoptosis profile, oxidative stress, mitochondrial network dynamics and basal adipogenic potential have been evaluated using various molecular and analytical techniques.

**Results:**

The SHBG knockdown altered the proliferative and metabolic activity of EqASCs, while dampening basal apoptosis via *Bax* transcript suppression. Furthermore, the cells treated with siRNA were characterized by senescent phenotype, accumulation of reactive oxygen species (ROS), nitric oxide, as well as decreased mitochondrial potential that was shown by mitochondrial membrane depolarization and lower expression of key mitophagy factors: PINK, PARKIN and MFN. The addition of SHBG protein reversed the impaired and senescent phenotype of EMS-like cells that was proven by enhanced proliferative activity, reduced apoptosis resistance, lower ROS accumulation and greater mitochondrial dynamics, which is proposed to be related to a normalization of *Bax* expression. Crucially, SHBG silencing enhanced the expression of key pro-adipogenic effectors, while decreased the abundance of anti-adipogenic factors namely HIF1-α and FABP4. The addition of exogenous SHBG further depleted the expression of PPARγ and C/EBPα and restored the levels of FABP4 and HIF1-α evoking a strong inhibitory potential toward ASCs adipogenesis.

**Conclusion:**

Herein, we provide for the first time the evidence that SHBG protein in importantly involved in various key metabolic pathways governing EqASCs functions, and more importantly we showed that SHBG negatively affect the basal adipogenic potential of tested ASCs through a FABP4-dependant pathway, and provide thus new insights for the development of potential anti-obesity therapeutic approach in both animals and humans.

**Graphical Abstract:**

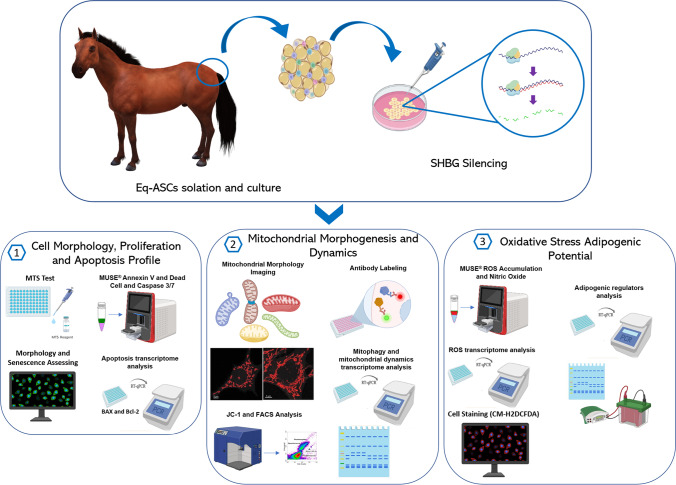

## Introduction

Sex hormone-binding globulin (SHBG) is a 90-100 kDa glycoprotein produced most abundantly in the liver, of which the primary physiological role is the transport of dihydrotestosterone (DHT), testosterone (T), estradiol (E2), estrogen, and other sex steroids [1]. Because of its spatial structure and biochemical functions, SHBG may modulate the concentration and bioavailability of biologically active sex hormones in the peripheral blood. Serum SHBG level is dependent on its glycosylation status, and a number of recent studies have shown serum SHBG correlation with obesity, type 2 diabetes, and other endocrine disorders [2], which makes it an interesting biomolecule from a diagnostic perspective. It was previously thought that body mass index is a critical determinant of SHBG level; however, liver fat deposition and liver lipids accumulation seem to play a pivotal role in the regulation of SHBG production and secretion [3]. Interestingly, db/db mice with overexpressed SHBG were resistant to obesity development, and fatty liver or hepatosteatosis [4, 5]. Besides, recent studies reported on the hormone-like effects of SHBG on proximal tubule epithelial cells [6], breast cancer [7], and our earlier studies demonstrated that SHBG modulates hepatocytes viability and lipolytic genes expression, and finally protects against ER stress [8]. The changes in systemic SHBG levels have additionally been proposed as a potential biomarker for both inflammatory disorders and metabolic syndrome [9]. Although the physiological role of SHBG is relatively well described, little is known about its impact on adipose tissue microenvironment and adipose stromal cells stemness.

Adipose-derived stromal cells (ASCs) belong to one of the most frequently used stem cells pool in the field of regenerative medicine. They are characterized by the expression of typical stromal cells surface antigens including CD34, CD90, CD106, and lack of CD45 expression, and as adherent, spindle shaped cells, they are capable to form clonogenic colonies and are characterized by elevated proliferation and expansion potential [10]. ASCs, as multipotent cells with self-renewal properties, become a unique population of stromal cells that can give rise to osteogenic, adipogenic, chondrogenic, and neurogenic cells and therefore modulate also various tissues’ microenvironments. The manner by which ASCs expand directly impacts adipogenesis and adipose tissue growth and remodeling and thus plays a critical role in adipose tissue inflammation and sensitivity to insulin [11].

In the past few decade, ASCs have been shown to be clinically useful for the regeneration of many different injured tissues in both human and veterinary medicine [12]. Their unique pro-regenerative potential has been identified in musculoskeletal system disorders treatment [13], neurological, and endocrinological implications including metabolic syndrome [14].

Nowadays, adipose tissue is no longer recognized as being strictly an energy storage tissue but is known to play a fundamental endocrine role in both physiology and pathology including lipids homeostasis, energy metabolism, obesity, and obesity-related metabolic disorders. Adipose tissue stromal cells niches due to their spatial organization may modulate the fate of other stroma residing cells, and existing clones’ multipotency [15]. Adipocyte stem and progenitor cells that are located around blood vessels and in adipose tissue microenvironment have been shown to play an important role in the modulation of adipose tissue hormonal balance and homeostasis; however, together with age or diseases, they are losing their unique pro-regenerative and immunomodulatory potential. Their multipotency or plasticity in response to unfavorable pro-inflammatory insults affect their differentiation potential including adipogenesis and therefore modulate adipose tissue inflammation and fibrosis [16]. Under equine metabolic syndrome (EMS) condition, the equine adipose tissue microenvironment has similarly been reported to exhibit altered metabolism and persistent pro-inflammatory phenotype, which strongly impairs the residing cells including ASCs [17]. Hence, previous studies have demonstrated that ASCs isolated from EMS-derived adipose tissue were characterized by profound biological failures with reduced transdifferentiation potential, viability, proliferation capacity, disrupted heterochromatin architecture and premature senescence [18, 19].

Abundant oxidative stress and hyperinsulinemia negatively impact ASCs’ multipotency by impairing mitochondrial biogenesis and dynamics [20]. In particular, mitochondrial biogenesis and dynamics have been shown to play an important regulatory role in MSCs “rejuvenation”, and defective mitochondrial transfer between stromal cells and macrophages is recognized as a critical mechanism initiating or inhibiting inflammation development. Accordingly, in EMS horses, ASCs have been reported to display critical altered mitochondrial metabolism. Formerly, Kornicka et al. [21], evidenced a loss in mitochondrial transmembrane potential, excessive accumulation of ROS, and deficient endogenous antioxidant defenses in EMS-derived ASCs. Moreover, the same study demonstrated prominent mitochondrial morphology abnormalities, associated aberrant fission and significantly impaired mitophagy, which has been underscored as an essential protective and recycling mechanism in MSCs. The defective mitochondrial biogenesis and dynamics in ASCs or beige adipocytes may play a pivotal role in altered adipogenesis and adipocyte hypertrophy, and thus may impact adipose tissue insulin sensitivity [22, 23]. While various adipokines and hepatokines have been reported to either promote or hamper ASCs functions, whether SHBG impacts the metabolic pathways governing ASCs fate, including survival, expansion, inflammation and mitochondrial biogenesis and dynamics are still unknown.

In this study, we investigated the molecular effects of SHBG on a model of equine ASCs and verified the outcomes of SHBG silencing on their expansion, survival, oxidative stress, mitochondrial dynamics and basal adipogenic potential. We presented the evidence of the important role of SHBG in the modulation of ASCs functions and the plausible antiadipogenic potential of the hepatokine.

## Materials and Methods

All chemicals and reagents used in the study were obtained from Sigma Aldrich (Poznań, Poland), unless otherwise stated. Cell culture reagents were purchased from BioWest (VWR International, Gdańsk, Poland).

### Equine Adipose-Derived Stromal Cells (ASCs)

The equine ASCs used in the study were obtained from a collection of the Department of Experimental Biology, University of Environmental and Life Sciences, Wroclaw, Poland. The cell isolation protocol and immunophenotyping have been described in previous works [24]. Secondary ACSs cultures were used at third passage and maintained in Dulbecco’s modified Eagle’s medium Low Glucose (DMEM Low Glucose) supplemented with 1000 mg/l glucose, 10% foetal bovine serum (FBS) and 1% of a penicillin and streptomycin (PS) solution, in standard condition (37 °C, 5% CO_2_).

### Sex Hormone Binding Globulin (SHBG) Silencing and Treatment

SHBG protein expression was silenced in ASCs using small interfering RNA (Silencer® Select Pre-designed siRNA, Ambion, exon 3) and Lipofectamine RNAiMAX Reagent (Invitrogen, Thermo Fisher Scientific) according to the manufacturer’s protocol. The concentration of used siRNA was equal to 20 nM.

All experiments used three study groups: (i) ‘HE ASC’ control cells (untreated), (ii) ‘HE ASC SHBG-’ SHBG suppressed cells, and (iii) SHBG suppressed cells incubated with SHBG protein at a concentration of 50 nM - ‘HE ASC SHBG- + SHBG’ (SHBG; Fitzgerald, 30R-AS012).

The course of the experiments described in the following sections was similar, namely, cells of all groups were seeded in appropriate vessels. After cells adhesion, the expression of SHBG gene was silenced for 72 hours. Then, the culture medium was changed to Dulbecco’s modified Eagle’s medium Low Glucose (DMEM Low Glucose) supplemented with 1000 mg/l glucose, 0.02% bovine serum albumin (BSA) and 1% of a penicillin and streptomycin (PS) solution, and, in the case of HE SHBG- + SHBG’, 50 nM SHBG protein. After a 24-hours incubation under standard conditions, the cells were prepared for analysis according to the procedures provided in the individual experiments. The final used SHBG concentration has been selected on the basis of cytotoxicity screening assays and projected from the literature data to reflect the median physiological levels of SHBG in both horses and humans [25, 26].

### Cells Viability

The viability of EqASCs cells in all three experimental groups was assessed by three different methods, namely by examining the metabolic activity of the cells, the expression level of the Ki67 cell proliferation marker and the wound healing assay.

- Determination of cell viability and metabolic activity was performed by MTS Cell Viability Assay (Abcam, ab197010) according to the manufacturer’s protocol. Briefly, cells were seeded on 24-well plate in fresh full medium. After incubation of the cells with the tested compounds (as described in “Point 2”), the medium was changed to fresh medium containing 10% MTS and the cells were cultured for 2 hours under standard conditions. After incubation the media were transferred onto 96-well plate and absorbance was measured spectrophotometrically at 490 nm (Epoch, Biotek, Bad Friedrichshall, Germany).
- The level of proliferation marker Ki67 was analysed using confocal microscopy. For this purpose, the cells were seeded on microscope coverslips placed in 24-well plates. After the experiment, the cells were fixed with 4% paraformaldehyde (PFA) for 20 minutes at room temperature (RT) and permeabilized in 0.2% Tween +10% goat serum for 20 minutes at RT. The cells were then incubated in the presence of the anti-Ki67 rabbit antibody (Abcam, ab15580) at a 1:1000 dilution, overnight at 4 °C (Table 1). A goat anti-rabbit-Atto 594 antibody was used as the secondary antibody at 1:1000 dilution (1 hour, RT). Three rinses of Phosphate-buffered saline (PBS) were used between each step. Finally, the coverslips were mounted onto microscope slides using the ProLong™ Diamond Antifade Mountant with DAPI (Invitrogen Life Technologies, Warsaw, Poland).

**Table 1:** The antibodies used for Western Blot and Immunocytochemistry

Antibody	Catalog No./ Origin	Dilution (WB)	Dilution (ICC)
PARKIN	Novus Biologicals, nbp2-67,017	1:250	1:200
MFN	Biobyrt, orb11040	1:500	1:200
PINK	Biorbyt, orb331223	1:250	0.5 μg/ml
KI67	Abcam, ab15580	–	1:1000
CEBPα	Aviva, ARP57937	0.5 mg/ml	–
FABP4	Aviva, ARP33794	0.5 mg/ml	–
GLUT-4	Abcam, ab33780	1 μg/ml	–
FASN	Affinity Biosciences, DF6106	1:500	–
SHBG	Biorbyt, Orb11366	1:1000	–
β-Actin	Sigma Aldrich, a2066	1:5000	–
Goat Anti-Rabbit IgG H&L (HRP)	Abcam, ab205718-500	1:10000	–
Rabbit Anti-Mouse IgG H&L (HRP)	Abcam, ab6728-1	1:10000	–
Goat Anti-Mouse IgG - Atto 594	Sigma, 76,085	–	1:1000
Goat Anti-Rabbit IgG - Atto 594	Sigma, 77,671	–	1:1000
  Microscopic preparations were analysed using confocal microscope (Leica DMi8, Leica Microsystems, KAWA.SKA Sp. z o.o., Poland) with a magnification of ×630. The photomicrographs were analysed in the Fiji is just ImageJ Software (version 1.52n, Wayne Rasband, National Institutes of Health, USA) using the ‘Colour Pixel Counter’ plugin (analysed thresholds: 49, 50 and 51).
- Wound healing assay was used to study cell migration and cell–cell interaction. Cells were seeded in a 24-well plate and treated as previously described. After obtaining a fully confluent monolayer of cells, a sterile pipette tip was used to scrape a wound across the entire centre of the well. The changing distance between the edges of the resulting ‘wound’ was analysed using the Leica DMi1 manual inverted microscope (Leica Microsystems) after 24 h and 48 h.

### Senescence Analysis

Cellular senescence was analysed using the β-galactosidase hydrolysis kit (Senescence Cells Histochemical Staining Kit, Sigma Aldrich) according to the manufacturer’s instructions. The cells were seeded in 24-well plates, the experiment was carried out and then the cells were incubated with the staining solution for 24 h. Microphotographs were taken at ×100 and ×400 magnification using the Leica DMi1 manual inverted microscope (Leica Microsystems). Colour analysis was performed in the Fiji is just ImageJ Software (version 1.52n, Wayne Rasband, National Institutes of Health, USA) using the ‘Colour Pixel Counter’ plugin (analysed thresholds 179, 180 and 181).

### Analysis of Viability and Apoptosis by Capillary Flow Cytometry

Metabolic changes of the examined cells were analysed by capillary flow cytometry using the Muse® Cell Analyzer (EMD Millipore Corporation) and the staining kits recommended by the producer, according to the manufacturer’s instructions.

- Cell viability and apoptosis were analysed using the Muse® Annexin V & Dead Cell Kit (Luminex). Approximately 1 × 10^5^ cells were suspended in 100 μl of Muse® 1X Assay Buffer and mixed with 100 μL of Muse® Annexin V & Dead Cell Reagent. Cells were incubated for 20 minutes in the dark at room temperature and then analysed using the MUSE instrument.
- Caspase activation and cellular plasma membrane permeabilization was evaluated using the Muse® Caspase-3/7 Kit (Luminex). Approximately 1 × 10^4^ of tested cells were suspended in 50 μl Muse® 1X Caspase buffer and mixed with 5 μl of Muse® MultiCaspase working solution. The cells were incubated for 30 minutes at 37 °C, 150 μl of Muse® 7-AAD working solution was added and cells were analysed using the MUSE apparatus.

### Analysis of Oxidative Stress and Nitric Oxide

Oxidative stress and nitric oxide profile in the examined cells were analysed by capillary flow cytometry using the Muse® Cell Analyzer (EMD Millipore Corporation) as described in ‘Section 2.5’:

- Oxidative stress was measured using the Muse® Oxidative Stress Kit (Luminex). Approximately 1 × 10^4^ of tested cells were suspended in 10 μl Muse® 1X Assay Buffer and mixed with 190 μl Muse Oxidative Stress working solution, incubated for 30 minutes at 37 °C and then analysed using the MUSE apparatus.
- Changes in intracellular nitric oxide activity levels were tested using the Muse® Nitric Oxide Kit (Luminex). Approximately 1 × 10^4^ of tested cells were suspended in 10 μl Muse® 1X Assay Buffer and mixed with 100 μl of Muse® Nitric Oxide working solution and incubated for 30 minutes at 37 °C. Then, 90 μl of Muse® 7-AAD working solution was added and finally cells were analysed using the MUSE apparatus.
- Furthermore, the reactive oxygen species were stained in cells using CM-H2DCFDA (General Oxidative Stress Indicator) (Invitrogen, C6827). The cells were stained accordingly to manufacturers’ instruction. The slides were closed using the ProLong™ Diamond Antifade Mountant with DAPI (Invitrogen Life Technologies, Warsaw, Poland). Microscopic preparations were observed using confocal microscope (Leica DMi8, Leica Microsystems, KAWA.SKA Sp. z o.o., Poland) with a magnification of ×630. The photomicrographs were finally analysed in the Fiji is just ImageJ Software (version 1.52n, Wayne Rasband, National Institutes of Health, USA).

### Mitochondrial Network Development

The analysis of the mitochondrial network was performed by fluorescent staining with MitoRed (Sigma Aldrich, Poznan, Poland) fluorescent dye (1:1000 in culture medium) for 30 min at 37 °C, prior to fixation in 4% paraformaldehyde at RT for 20 min. In addition, actin cytoskeleton was stained for 40 min at 37 °C using solution of Phalloidin-Atto 488 (Sigma Aldrich, Munich, Germany) in the concentration 1:800. ProLong™ Diamond Antifade Mountant with DAPI (Invitrogen™, Warsaw, Poland) was used for nuclei staining. The stained cells were observed using confocal microscope (Leica DMi8, Leica Microsystems, KAWA.SKA Sp. z o.o., Poland) at a magnification of ×1000. The mitochondria morphology was then assessed in the MicroP software (analysed thresholds: 1, 1.5, 2.0). In addition, the mitochondria staining intensity was analysed in the Fiji is just ImageJ Software (version 1.52n, Wayne Rasband, National Institutes of Health, USA) using the ‘Colour Pixel Counter’ plugin (analysed thresholds: 49, 50 and 51).

### Mitochondrial Transmembrane Potential

The mitochondrial potential (ΔΨm) was tested using the MitoProbe™ JC-1 Assay Kit for Flow Cytometry (M34152, Molecular Probes, Life Technologies) according to the manufacturer’s instructions. Stained cells were analysed by confocal microscopy and fluorescence-activated cell sorting technique (FACS):

- For microscopy, cells were rinsed with PBS and JC-1 solution (2 μM of JC-1 in full medium) was added to the samples. The samples were incubated for 30 minutes at 37 °C, then cells were fixed in 4% PFA and slides were mounted using ProLong™ Diamond Antifade Mounting with DAPI. Pictures were acquired using a confocal microscope (Leica DMi8, Leica Microsystems, KAWA.SKA Sp. z o.o., Poland) at a magnification of ×630.
- For FACS analysis, cells in suspension were stained in an analogous way with JC-1 solution, resuspended in 500 μl of PBS and analysed in BD LSRFortessa™ Cell Analyzer (Becton Dickinson, Franklin Lakes, New Jersey, USA). Detectors used for the analysis: PE and FITC. Except staining with JC-1 solution, a control groups were additionally treated for 5 min at 37 °C with CCCP solution (50 μM in full medium) that was supplied with the kit. The purpose of CCCP treatment is to confirm that JC-1 response is sensitive to changes in membrane potential.

### Protein Expression Profiling

The level of proteins related to mitochondrial function, as well as adipogenesis were analysed by both Western blot and immunofluorescence staining.

- Cells for confocal microscopy were prepared as previously described (overnight incubation at 4 °C) using antibodies directed against PARKIN, MFN and PINK proteins with simultaneous staining of cell nuclei with DAPI. The concentration of each antibody was presented in Table 1. The secondary antibodies were Atto 594 - conjugated and produced in goat (anti-mouse or anti-rabbit) and added to the samples at a final concentration of 1:1000. The samples were incubated with secondary antibodies for 1 h at room temperature.
- For Western Blot Analysis, EqASCs cells were collected and homogenized in RIPA lysis buffer with phosphatases and proteases inhibitors cocktail for 2 hours on ice. Proteins were collected by centrifugation of cell lysates for 20 min at 4 °C and 6000×g and the concentration was determined using the Pierce™ Bicinchonic Acid (BCA) Protein Assay Kit. Protein samples were diluted in Laemmli Loading Buffer (Bio-Rad, Hercules, CA, USA) and denatured at 95 °C for 5 min. SDS-polyacrylamide gel electrophoresis was performed for 90 min in Tris/glycine/SDS buffer in Mini-PROTEAN Tetra vertical electrophoresis cell (Bio-Rad, Hercules, CA, USA), followed by protein transfer on polyvinylidene difluoride (PVDF) membrane (Bio-Rad, Hercules, CA, USA) with a Mini Trans-Blot®Cell transfer apparatus (Bio-Rad, Hercules, CA, USA) in Tris/glycine/methanol buffer with 100 V, 250 mA at 4 °C for 45 minutes. After transfer, membranes were blocked in 5% skim milk solution in TBST for 1 h at room temperature, then incubated overnight at 4 °C in each corresponding primary antibody (Table 1) and secondary antibodies conjugated to HRP (dilution 1: 2500 in TBST) for 1 h at room temperature. Chemiluminescence analysis was performed using the ChemiDoc MP imaging system (Bio-Rad, Hercules, CA, USA) and quantified by Image Lab software (Bio-Rad, Hercules, CA, USA).

### Real-Time Reverse Transcription PCR (qRT-PCR)

For qRT-PCR analysis, total RNA was extracted from tested cells using TRIzol reagent, according to the manufacturer’s instructions. The purity and concentration of obtained RNA were measured by a nanospectrophotometer (Epoch, Biotek, Bad Friedrichshall, Germany). cDNA was then prepared from 500 ng of RNA using PrimeScript™ RT reagent kit with gDNA eraser (TaKaRa, Gdansk, Poland) in a T100 thermal cycler (Bio-Rad, Hercules, CA, USA) according to the manufacturer’s instructions.

RT-qPCR analysis was performed using the SensiFAST SYBR Green Kit (Bioline, London, UK) on the CFX Connect™ real-time PCR detection system (Bio-Rad). Briefly, 5 μl of SensiFAST SYBR Master mix was combined with 2.5 μl (final concentration equals to 400 nM) of targeted primer and 2.5 μl of obtained cDNA. Thermal cycle conditions were: 95 °C for 2 min, followed by 40 cycles at 95 °C for 15 s, annealing for 15 s at the temperature specified for the primers, and elongation at 72 °C for 15 s. The results are given in terms of Glyceraldehyde-3-phosphate dehydrogenase (GAPDH) expression. Relative gene expression was calculated for all study groups using the RQ_MAX_ method and presented in a log scale. Sequences for all primers used are listed in Table 2.

**Table 2:** The primers’ sequences used in RT-qPCR

Gene	Primer	Sequence	Amplicon length	Accession number
*Parkin*	F:	CTGGAGGATTTAGTCCCGGAGC	138	XM_005608125.3
	R:	CCATGGCTGGAGTTGAACCTG		
*Mfn*	F:	AATGCCATGCTCTGGGACAA	325	XM_023635773.1
	R:	CATCAGCGTCCAGGCAAAAC		
*Pink*	F:	GCACAATGAGCCAGGAGCTA	298	XM_014737247.2
	R:	GGGGTATTCACGCGAAGGTA		
*Mief1*	F:	ATGCTGGGCATCGCTACAC	284	XM_023631522.1
	R:	CGGAGCCGTGACTTCTTCAA		
*Mief2*	F:	CGTTCTATTATCAGGCAGGTCC	108	XM_005597824.3
	R:	AGAACTCTGCCATGGTCTTCT		
*Miro1*	F:	GATCCTGCTGGTGGGAGAAC	88	XM_023651639.1
	R:	GGGAGGAACCTCTTCTGGGA		
*Pgc1a*	F:	GGCCTTCTAAACGTGGGACA	135	XM_023630977.1
	R:	CCGGAGGTCTGCCATTTTCT		
*Bax*	F:	CGAGTGGCAGCTGAGATGTT	153	XM_023650077.1
	R:	AAGGAAGTCCAGTGTCCAGC		
*Bcl2*	F:	TTCTTTGAGTTCGGTGGGGT	164	XM_014843802.1
	R:	GGGCCGTACAGTTCCACAA		
*Gapdh*	F:	GATGCCCCAATGTTTGTGA	250	NM 001163856.1
	R:	AAGCAGGGATGATGTTCTGG		
*Sod1*	F:	CATTCCATCATTGGCCGCAC	130	NW_001867397.1
	R:	GAGCGATCCCAATCACACCA		
*Sod2*	F:	GGACAAACCTGAGCCCCAAT	125	NW_001867408.1
	R:	TTGGACACCAGCCGATACAG		
*Gpx*	F:	TCGAGCCCAACTTCACACTC	178	NM_001166479.1
	R:	AAGTTCCAGGCGACATCGTT		
*Cat*	F:	ACCAAGGTTTGGCCTCACAA	112	XM_014851065.1
	R:	TTGGGTCAAAGGCCAACTGT		
*Ppar*γ	F:	TTTCGCTCAGTGGAAGCTGT	191	XM_014846252.1
	R:	GGAGGCCAGCATGGTGTAAA		
*Cebpa*	F:	TCCCGGAGGGACCAAAGTTA	116	XM_023649498.1
	R:	CTCACATTGCACAAGGCACC		
*Glut-4*	F:	TTTGTGGCATTCTTTGAGA	65	NM_001081866.2
	R:	CTGAAGAGCTCAGCCACG		
*Fasn*	F:	CCCCACAGCTACACTTCCAG	326	XM_023651718.1
	R:	CCGAGCAGGGTTGGATCTTT		
*Srebp1c*	F:	TCAGCGAGGCGGCTTTGGAGCAG	80	XM_008542859.1
	R:	CATGTCTTCGATGTCGGTCAG		
*Acly*	F:	CCACTTCAGAGCCCAGACAA	361	XM_005597396.3
	R:	AACTAGGCCCAGCTTTCCAC		
*Hif1a*	F:	CTCAAATGCAAGAACCTGCTC	108	LOC100061166
	R:	TTCCATACCATCTTTTGTCACTG		

*Parkin* PRKN parkin RBR E3 ubiquitin protein ligase, *Mfn1* Mitofusin 1, *Pink* PTEN-induced kinase 1, *Mief1* Mitochondrial elongation factor 1, *Mief2* Mitochondrial elongation factor 2, *Miro1* Mitochondrial *Rho GTPase 1*, *Pgc1a* Peroxisome proliferator-activated receptor gamma coactivator 1-alpha, *Bax* BCL2 Associated X, *Bcl2* B cell lymphoma 2, *Gapdh* Glyceraldehyde-3-phosphate dehydrogenase, *Sod1* Superoxide dismutase type 1, *Sod2* Superoxide dismutase type 1, *Gpx* Glutathione Peroxidase, *Pparγ* Peroxisome proliferator- activated receptor gamma, *Cebpa* Curved DNA binding protein A, *Glut-4* Glucose transporter type 4, *Fasn* Fatty acid synthase, *Srebp1c* Sterol regulatory element-binding transcription factor 1, *Acly* ATP Citrate Lyase, *Hif1a* Hypoxia-inducible factor 1-alpha

### Statistical Analyses

Results were presented in individual data graphs with mean ± SD. The statistics were performed using GraphPad Prism 8 (GraphPad Software, San Diego, CA, USA) by the use of one-way analysis of variance (ANOVA) with Tukey’s post-hoc test. Each experimental assay was performed at least in three replicates. Differences were considered as statistically significant at p < 0.05. The significance levels were indicated with asterisks: *p < 0.05, **p < 0.01, ***p < 0.001 and ****p < 0.0001.

## Results

### SHBG Knockdown Efficiency

**Fig. 1:**
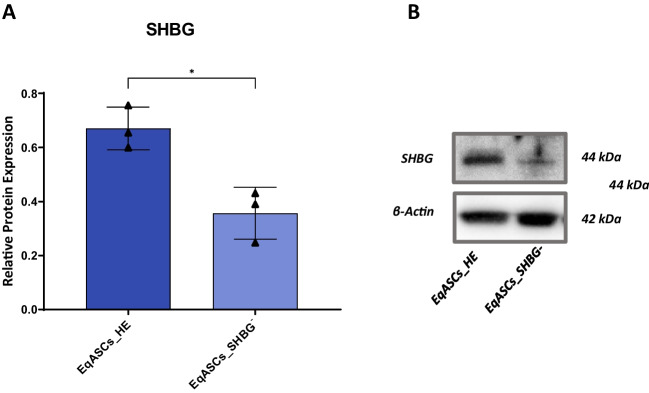
SHBG silencing efficiency (**A**). Protein expression was detected using Western blot technique (**B**). Significant differences were calculated for normalized values and shown as means ± SD. *p value < 0.05

In order to evaluate the efficiency of SHBG silencing on EqASCs, the expression of SHBG protein was analyzed by the Western Blot method. As shown in the Fig. 1, application of SHBG siRNA to ASCs resulted in a significant downregulation of SHBG protein when compared to native control cells (*p < 0.05*). A reduced SHBG level instead of a total suppression has been established as an experimental model which mimics the observed status of SHBG in human clinical patients diagnosed for metabolic syndrome, and thus more realistically reflects the metabolic abnormalities linked to a drop in SHBG production.

### SHBG Silencing Induce Changes in Cell Morphology and Proliferation Profile

**Fig. 2:**
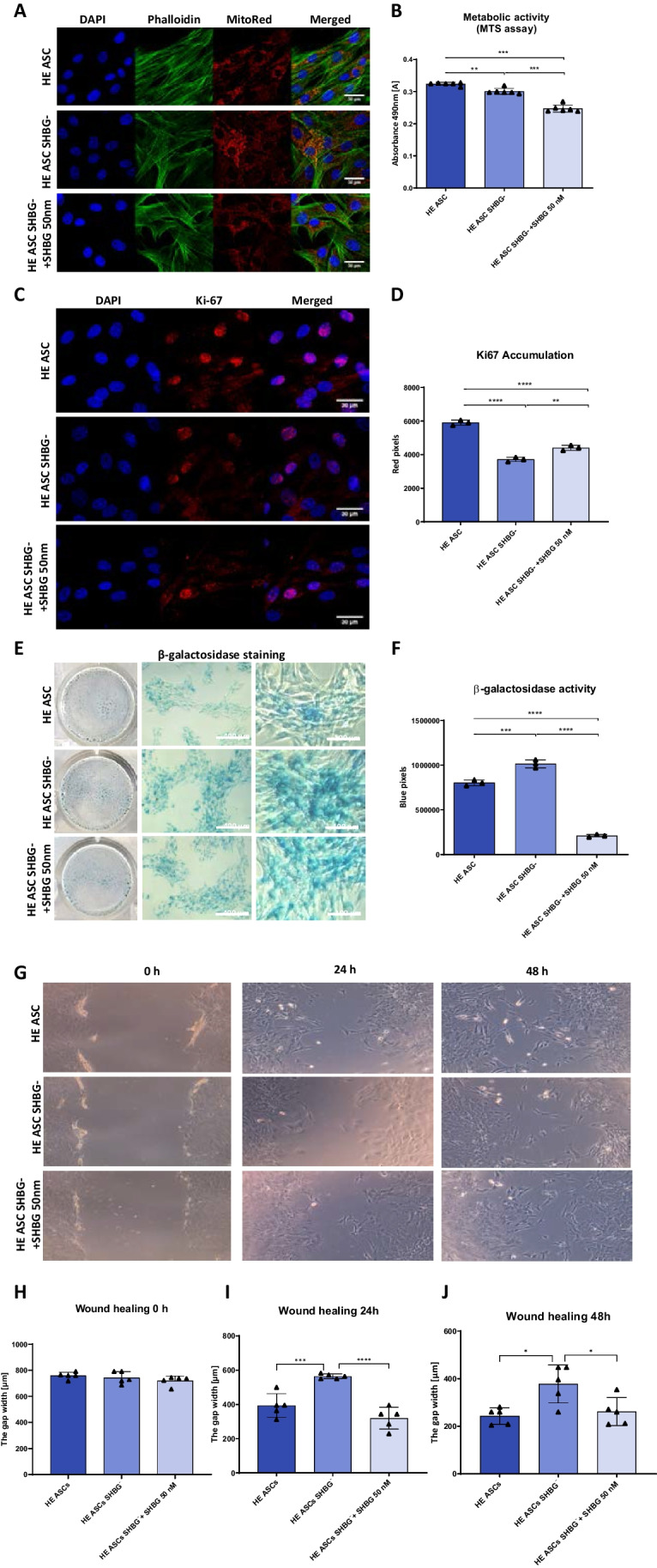
Morphology and viability of native, SHBG-silenced and after exogenous SHBG treatment. Morphology was evaluated under a confocal microscope of stained cells with – DAPI for cell nuclei, − phalloidin for F-actin cytoskeleton and mitoRed for mitochondrion (**A**). Metabolic activity was determined using MTS assay. The unit of metabolic activity factor was determined for silenced and treated cells, in reference to the native cells (considered as 1 = 100% of metabolic activity) (**B**). The proliferative capacity evaluated based on Ki67 expression and accumulation (**C**, **D**). Cell senescence was checked by β-galactosidase staining (**E**, **F**). Wound healing test (**G**-**J**). Bars representing means ± SD. *p value < 0.05, **p value < 0.01, ***p value < 0.001 and ****p value < 0,0001

To evaluate the implication of SHBG in cellular dynamics, changes in morphology and proliferation rate were analysed following SHBG silencing and after exogenous SHBG supplementation (Fig. 2A). Obtained results showed that after SHBG silencing, ASCs displayed irregular polygonal flattened geometry and enlarged size compared to native cells. Additionally, they lost their fibroblastic spindle-like shape. In addition, no significant changes in the cytoskeleton organization were noticed, as F-actin fibers appeared well defined and structured. Nuclei were round, prominent, and centrally placed without any signs of apoptosis-related fragmentation.

Next, the MTS assay was used to evaluate cell metabolic activity (Fig. 2B). SHBG silencing resulted in a significant decrease in the metabolic activity of cells compared to native cells (*p < 0,01*). Interestingly, treatment with exogenous SHBG further reduced the number of metabolically active cells when compared to both native (*p < 0,001*) and silenced cells (*p < 0,001*). By contrast, Ki67 staining showed opposite results as evidenced by the analysis of the marker of actively proliferating cells (Fig. 2C, D). Ki67 accumulation was significantly reduced in the SHBG silenced cells (*p < 0,001*), while incorporation of exogenous SHBG increased the proliferation rate compared to silenced cells (*p < 0,01*), which however remained significantly lower when compared to native cells (*p < 0,001*).

Premature cellular senescence was checked by measuring β-galactosidase activity (Fig. 2E, F). The obtained results showed that SHBG silencing increased the number of cells with highly active β­galactosidase as an evidence of cellular senescence establishment compared to native cells (*p < 0,001*). However, treatment of the silenced cells with SHBG significantly reduced senescence-positive cells both relative to native (*p < 0,0001*) and silenced cells (Fig. 2F; *p < 0,0001*).

To examine how silencing and SHBG treatment affect tissue regeneration, a wound healing test was conducted (Fig. 2G-J). The obtained data showed that SHBG knockdown significantly delayed wound healing compared to native cells. The wound gap appeared significantly greater in cells silenced at both 24 (Fig. 2I; *p < 0,001*) and 48 hours (Fig. 2J; *p < 0,5*) of the test compared to native cells. However, the treatment of knockdown cells with exogenous SHBG visibly increased the migratory and expansion capacity of cells, which was comparable to that of native cells, indicating that SHBG is necessary for proper ASCs proliferation, expansion and migration, as well as for efficient tissue regeneration after wounding.

### SHBG Knockdown Impairs Basal Apoptosis in EqASCs

**Fig. 3:**
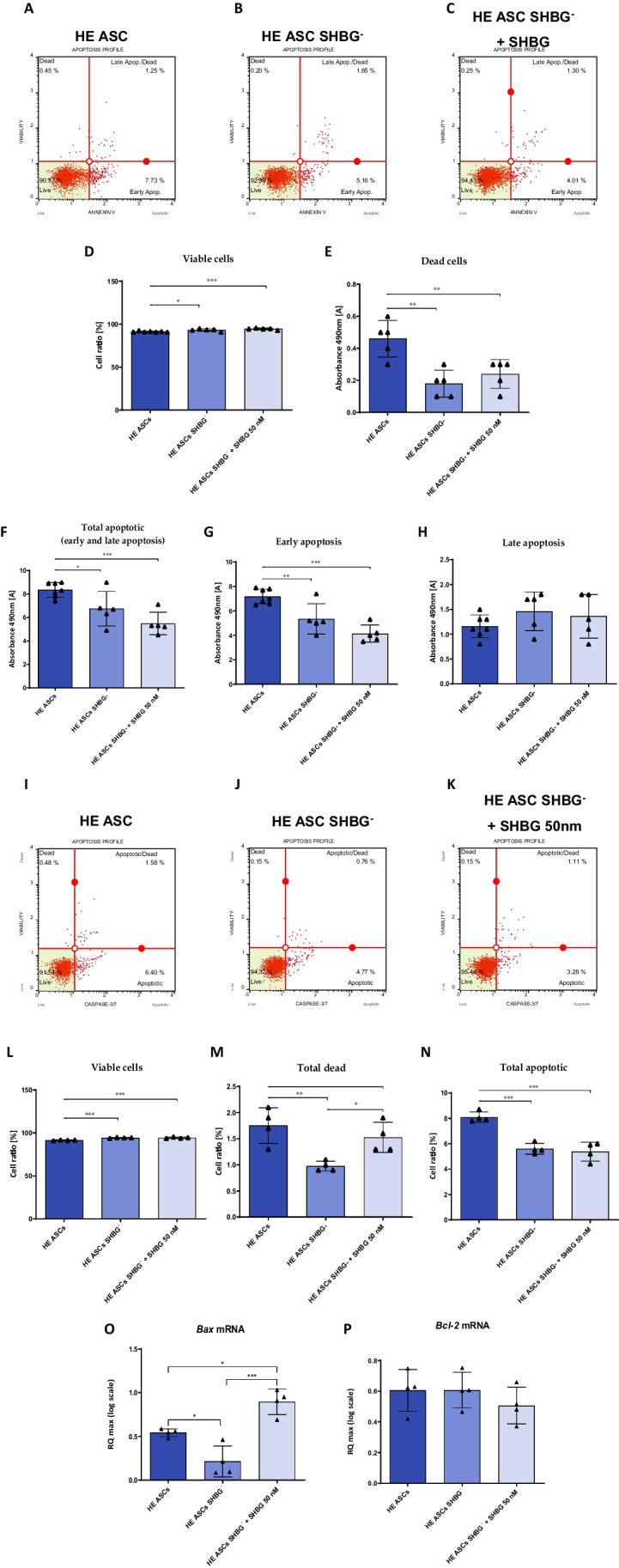
Impact of SHBG downregulation on apoptosis machinery. Apoptosis profile plots (**A**-**C**). Bar graphs showing the percentage of viable (**D**) and dead (**E**) cells and total apoptotic (**F**), early apoptotic (**G**), and late apoptotic (**H**) cells. Caspase-3/7 profile plots (**I**-**K**). Bar graphs depicting the percentage of viable (**L**), dead (**M**) and total apoptotic (**N**) cells. Representative bar graphs of the relative expression of apoptotic key markers *Bax* (**O**) and *Bcl-2* (**P**). Representative data are shown as mean ± SD. *p value < 0.05, **p value < 0.01 and ***p value < 0.001

The impact of SHBG loss on apoptosis was evaluated using the Muse^®^ Annexin V and Dead Cell (Fig. 3A-C) and Muse^®^ Caspase-3/7 Kit (Fig. 3I-K) assay kits for total apoptotic cell determination and caspase activation testing. The obtained results showed that the percentage of viable cells is slightly higher in the silenced cells culture compared to the native cells culture (*p < 0,05*), which was maintained after treatment of the cells with exogenous SHBG (Fig. 3D). Consistent with this, the percentage of dead cells in SHBG-silenced cells culture has been found significantly lower compared to the control group (*p < 0,01*), and remained unchanged after exogenous SHBG supplementation (Fig. 3E). SHBG-silenced cells similarly showed visible lower percentage of total apoptosis cells (including early and late apoptosis) compared to control cells (Fig. 3F; *p < 0,05*). This decrease, was however related to a significant difference in the number of apoptotic cells in early apoptosis, where a significant difference was noted (Fig. 3G; *p < 0,01*). Therewith, treatment of SHBG-silenced cells with exogenous SHBG did not alter the apoptotic profile in either its early or late stages as compared to silenced cells, and the number of cells in early apoptosis was additionally lower than in native cells (Fig. 3G, H).

To further elucidate the implication of SHBG in apoptotic events, activation of caspases 3 and 7 has been analysed. As presented in the Fig. 3I-N, SHBG silencing resulted in a lower percentage of apoptotic cells compared to native culture (*p < 0,001*), and treatment with exogenous SHBG did not significantly change this level (Fig. 3N).

In order to determine whether SHBG affects the expression of master apoptosis-regulators, gene expression of the BAX/BCL2 axis has been established. As shown in the Fig. 3O, the relative *Bax* transcript level was found to be critically downregulated after SHBG silencing when compared to native cells (*p < 0,05*). Moreover, an evident upregulation of the same transcript level was noted in SHBG-rescued cells, by opposition to both SHBG-depleted cells (*p < 0,001*) and native control group (*p < 0,05*). Next, the determination of relative *Bcl-2* transcript level showed no significant changes among the three experimental groups i.e., native, SHBG-silenced, and SHBG-rescued cells (Fig. 3P).

### SHBG Downregulation Triggers Excessive Oxidative Stress in EqASCs

**Fig. 4:**
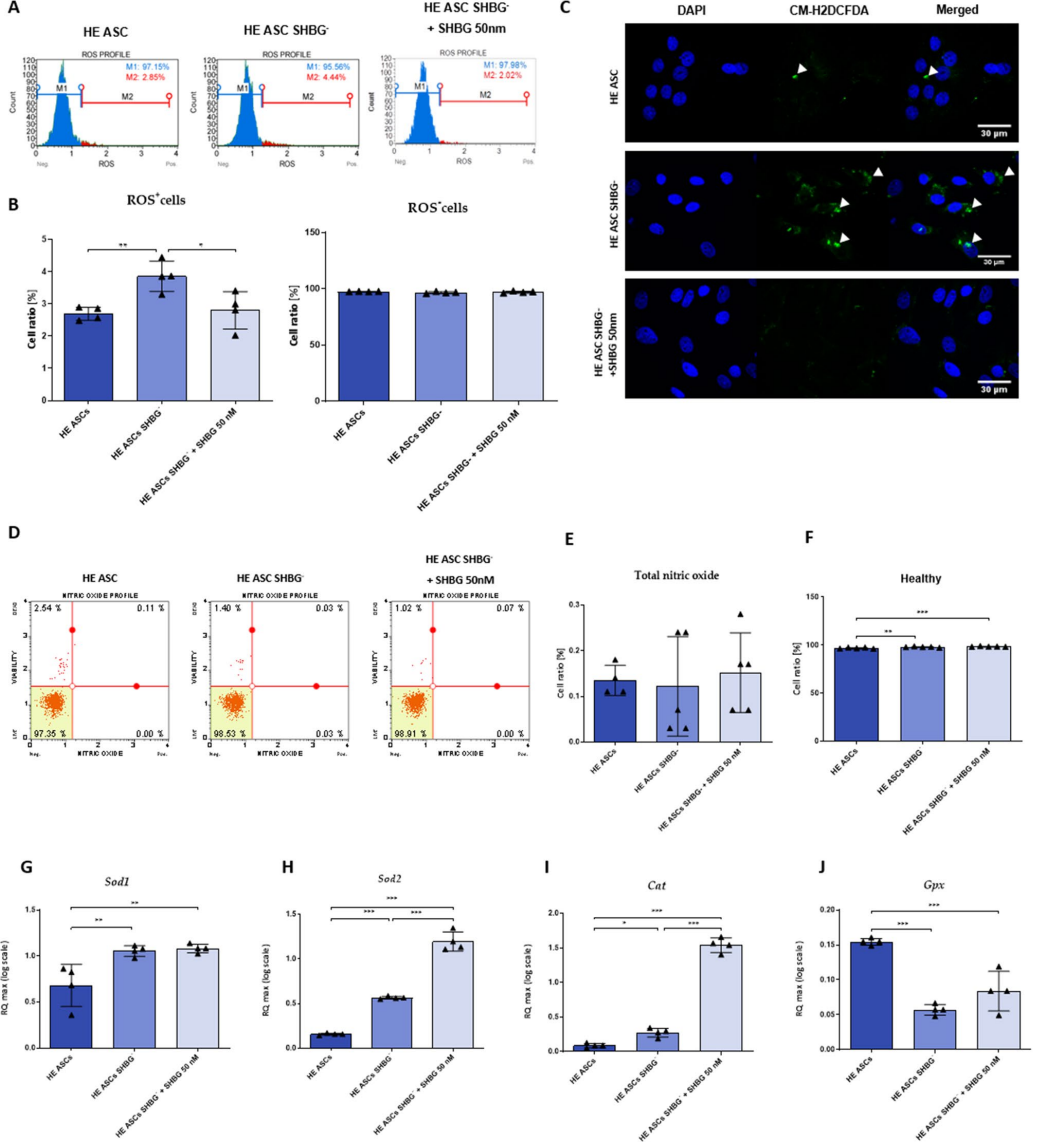
Evaluation of the oxidative status under SHBG deficiency condition. Histograms showing the distribution of cells based on ROS accumulation (**A**) and comparative analysis of ROS^+^ and ROS^−^ cells (**B**). ROS cells labeled with CM-H2DCFDA staining and analyzed by confocal microscopy (**C**). Representative plots showing the distribution of cells based on intracellular accumulation of nitric oxide (**D**) and statistical analysis of total nitric oxide (E) and healthy (**F**) cells. Graphs showing the relative expression of oxidative stress markers *Sod1* (**G**), *Sod2* (**H**), Cat (**I**) and *Gpx* (**J**). Representative data are shown as mean ± SD. *p value < 0.05, **p value < 0.01 and ***p value < 0.001

Oxidative stress levels following SHBG silencing were assessed by analyzing intracellular ROS accumulation using a Muse® Oxidative Stress Kit. (Fig. 4A, B). The silencing of SHBG induced excessive intracellular ROS production, as evidenced by the high accumulation of silenced cells in the ROS-positive cell population quadrant when compared to native cells (Fig. 4B; *p < 0,01*). Treatment of silenced cells with exogenous SHBG substantially reduced the percentage of ROS-positive cells (*p < 0,05*), which appeared comparable to that observed within the native cells group. These results were furthermore confirmed by the analysis of the fluorescence ROS indicator CM-H2DCFDA accumulation using confocal microscopy (Fig. 4C). The results confirmed an increase in the ROS intracellular levels within SHBG-depleted cells and a sensible decrease of ROS signal after the application of exogenous SHBG protein.

In similar fashion, SHBG involvement in modulating nitric oxide (NO) synthesis has been determined (Fig. 4D-F). The obtained results evidenced no critical impact of SHBG knockdown on NO levels when compared to native cells. In addition, the analysis of rescued cells showed no significant differences compared to both silenced and native cells (Fig. 4E).

In order to determine whether SHBG affects the expression of master genes involved in endogenous antioxidant responses, gene expression of *Sod1*, *Sod2*, *Gpx* and *Cat* has been established. The relative superoxide dismutase (*Sod1* and *Sod2*) transcript levels were found to be significantly upregulated after SHBG silencing by contrast to native cells (Fig. 4G, H; *Sod1*: *p < 0,01*, *Sod2*: *p < 0,001*), evoking the initiation of cellular antioxidant events in response to increased ROS generation. However, SHBG treatment did not change the level of cytoplasmic *Sod*, while mitochondrial *Sod2* appeared significantly upregulated relative to silenced and native cells (*p < 0,001*).

A similar effect was observed for the adaptive antioxidant enzyme *Cat* (Fig. 4I). Upregulation of its gene was observed after SHBG silencing (*p < 0,05*), and pronounced increase in the same relative transcript level was noted in SHBG-treated silenced cells compared to native untreated silenced cells (*p < 0,001*). Opposite trends were observed in terms of *Gxp* gene expression (Fig. 4J). SHBG-knockdown ASCs cells were characterized by a substantial suppressed *Gpx* expression when compared to native cells (*p < 0,001*), while SHBG-treated cells did not display any improved expression of the same gene. These data suggest the important role of SHBG in attenuating oxidative stress that maybe associated with a mitochondrial-mediated mechanism.

### Loss of SHBG Protein Does Not Affect Mitochondrial Morphogenesis but Strongly Reduces Transmembrane Potential

**Fig. 5:**
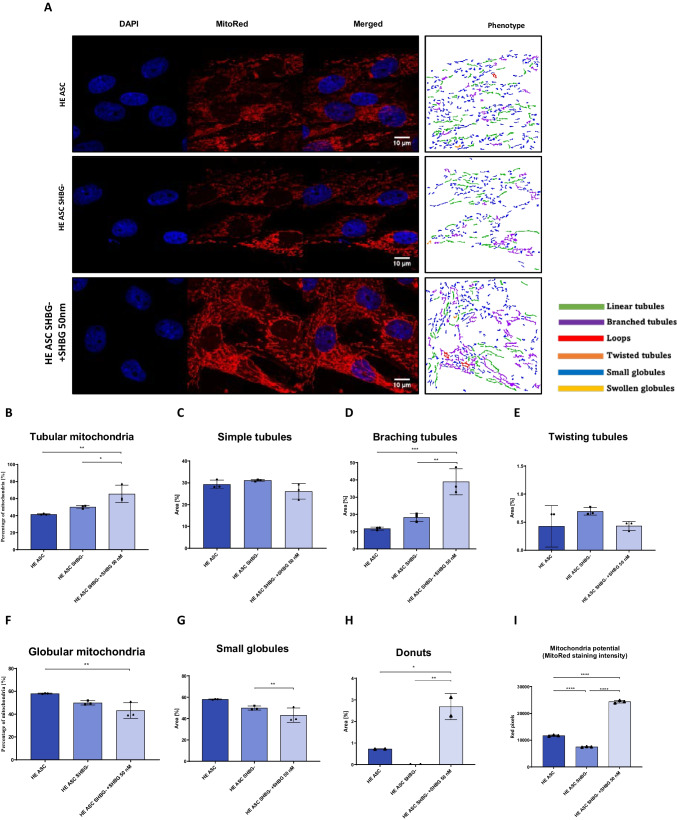
Analysis of mitochondrial network organization and transmembrane potential in the absence and presence of SHBG protein (**A**), classification of mitochondrial morphology (**B**-**H**) and analysis of mitochondrial network and membrane potential (**A**) and classification of mitochondrial morphology (**B**-**H**). Means ± SD as columns and bars. *p value < 0.05, **p value < 0.01, ***p value < 0.001 and ****p value < 0.0001

In order to verify whether SHBG metabolic effects are associated to the cellular mitochondrion, various mitochondrial health-related parameters have been evaluated. Mitochondrial morphology in native, SHBG-silenced, and SHBG-treated silenced cells was analyzed using the MicroP software (Fig. 5A). The obtained quantitative data showed that SHBG silencing did not result in significant changes in mitochondrial morphology (Fig. 5B-H). However, in the silenced cells treated with SHBG, an increase in the amount of tubular, branching, and donuts mitochondria (Fig. 5B, D, H) and a decrease in globular small mitochondria (Fig. 5G) was observed.

In addition, the mitochondrial membrane potential was measured using mitoRed staining. As shown in graph 4I, the mitochondria of silenced cells exhibited a drastically lower transmembrane potential compared to native cells (*p < 0,0001*). Treatment of silenced cells with the SHBG protein resulted in a significant increase in mitochondrial potential. This level appeared visibly higher than in silenced cells (*p < 0,0001*), but also exceeded the threshold level of native cells (Fig. 5I; *p < 0,0001*).

**Fig. 6:**
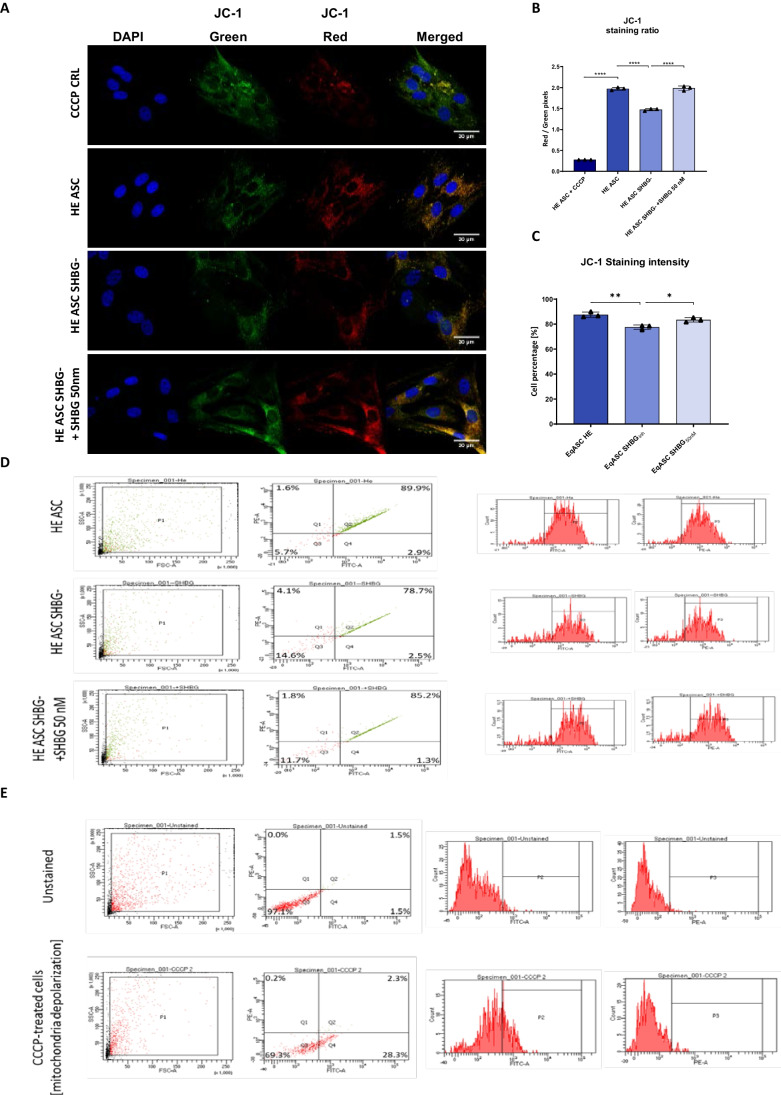
Impact of SHBG knockdown on potential and depolarization of the mitochondrial membrane. Analysis was performed using a confocal microscope using the specific JC-1 Red and JC-1 Green stains (**A**) and the results are presented in the form of a staining quantification graph (**B**). JC-1 staining was also evaluated using the FACS method (**C**-**E**). Representative data are shown as mean ± SD. *p value < 0.05, **p value < 0.01, and ****p value < 0.0001

In addition, JC-1 staining and FACS analysis were used to assess the potential of the mitochondrial membrane and its depolarization extend (Fig. 6). The formation of red JC-1 aggregates indicates a high potential of the mitochondrial membrane, while a low potential is characterized by a diffuse green fluorescent signal. The obtained results showed that silencing SHBG resulted in a significant abundance of depolarized mitochondria and resulting decreased mitochondrial membrane potential compared to the native cells. This is indicated by the analysis of microscopic photographs (Fig. 6A, B), and FACS analysis (Fig. 6C-E). Treatment of silenced cells with exogenous SHBG triggered a substantial restoration of mitochondrial membrane potential and simultaneous mitigation of depolarization to a basal level as compared to both control groups.

### SHBG Protein Depletion Disrupts Mitophagy and Mitochondrial Dynamics in EqASCs

To better investigate the relationship between SHBG protein and mitochondrial dynamics and function, parkin RBR E3 ubiquitin-protein ligase (PARKIN), PTEN-induced kinase 1 (PINK1) and mitofusin 1 (MFN1) expression analysis were performed at mRNA and protein level.

**Fig. 7:**
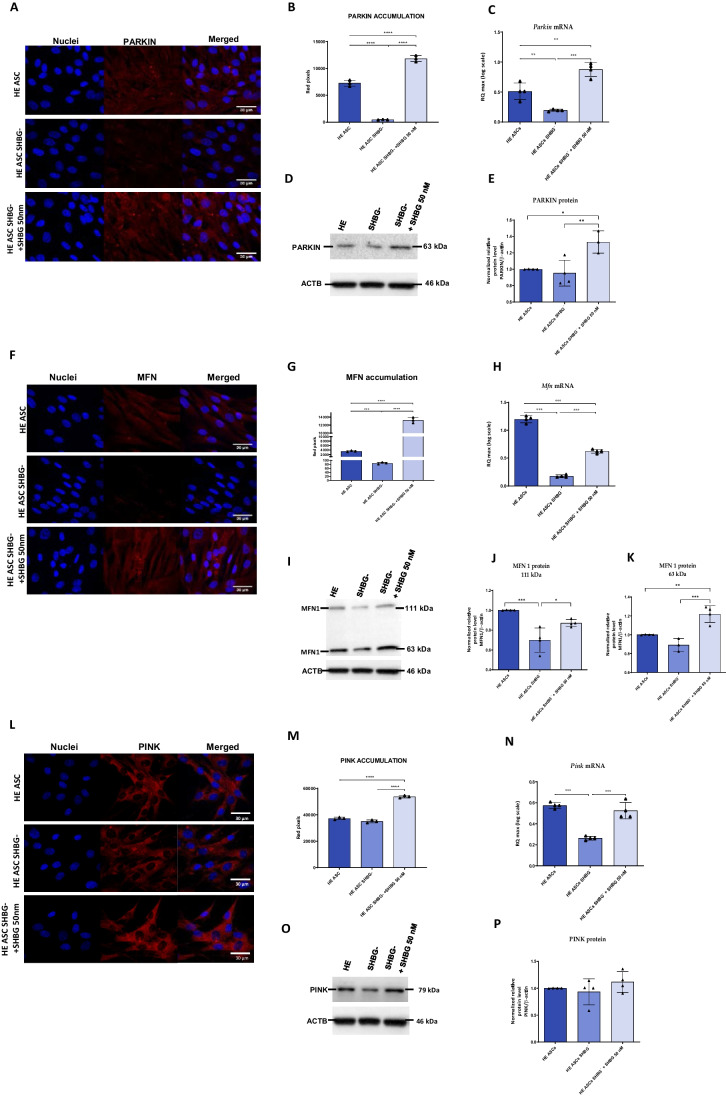
Changes in mitochondrial dynamics and mitophagy regulators expression following SHBG downregulation. PARKIN (**A**-**E**), PINK1 (**F**-**K**) and MFN1 (**L**-**P**) were determined based on antibody labeling analyzed by confocal microscope (**A**, **B**, **F**, **G**, **L**, **M**), mRNA (**C**, **H**, **N**) and protein levels (representative blots **C**, **D**, **I**-**K**, **O**, **P**). Representative data are shown as mean ± SD. * p value < 0.05, **p value < 0.01, ***p value < 0.001 and ****p value < 0.0001

The obtained quantitative data from immunofluorescence staining indicated that SHBG silencing resulted in a significant reduction of the PARKIN protein compared to native cells (Fig. 6A, B; *p < 0.0001*). In cells treated with exogenous SHBG, an increase in the accumulation of this protein was observed (Fig. 7A, B), both in relation to silenced cells (*p < 0.0001*) and native cells (*p < 0.0001*). Analysis of the *Parkin* gene relative expression level showed a downregulation in silenced cells compared to native cells (Fig. 7C; *p < 0.01*). In SHBG-silenced cells after exogenous SHBG treatment, a significant increase in expression of this gene was subsequently observed (Fig. 7C), both in relation to silenced cells (*p < 0.001*) and native cells (*p < 0.01*). Interestingly, analysis of PARKIN protein levels by Western Blot showed that SHBG knockdown did not result in any detectable expression changes (Fig. 7D, E). However, silenced cells treated with exogenous SHBG showed a significant increase in the level of this protein (Fig. 7D, E) both compared to silenced untreated cells (*p < 0.01*) and native cells (*p < 0.5*).

Subsequently, microscopic analyzes of MNF1 protein accumulation showed that silenced cells had a lower level of this protein than native cells (Fig. 7F, G; *p < 0.001*). Silenced cells treated with exogenous SHBG showed a significant increase in MNF1 accumulation (Fig. 7F, G) when compared to both SHBG-depleted cells (*p < 0.0001*) and native cells (*p < 0.0001*). A similar result was obtained when analyzing the relative expression level of *Mnf1* mRNA (Fig. 7H). SHBG-silenced cells had significantly downregulated *Mnf1* expression compared to native cells (*p < 0.001*). The silenced cells treated with exogenous SHBG showed upregulated relative to the silenced cells (*p < 0.001*), but was still at a lower level than in the native cells (*p < 0.001*). Western blot analysis showed that in silenced cells, the level of the 111 kDa MFN1 subunit was significantly reduced in regard to native cells (Fig. 7I, J; *p < 0.001*), while in SHBG-silenced cells treated with exogenous SHBG, its expression was found higher than in untreated knockdown cells (*p < 0.5*), but comparable to the level of native cells. On the other hand, the level of the 63 kDa subunit remained unchanged in silenced cells (Fig. 7I, K), however appeared elevated in cells treated with exogenous SHBG, both relative to silenced cells (*p < 0.001*) and native cells (*p < 0.01*).

Next, the expression level of PINK was analyzed. Immunofluorescence analysis showed that SHBG silencing did not result in significant changes in PINK protein accumulation (Fig. 7L, M). A significant increase over both silenced (*p < 0,0001*) and native cells (*p < 0,0001*) was noted in the silenced cells treated with exogenous SHBG. Further analysis showed that the *Pink1* gene was downregulated in silenced cells when compared to native cells (Fig. 7N; *p < 0.001*). Cells treated with SHBG showed an upregulation in *Pink1* mRNA level compared to deficient cells (*p < 0.001*), which was comparable to native cells. Analysis of the protein level by Western Blot showed no differences in both SHBG-silenced and exogenous SHBG-treated cells (Fig. 7O, P).

**Fig. 8:**
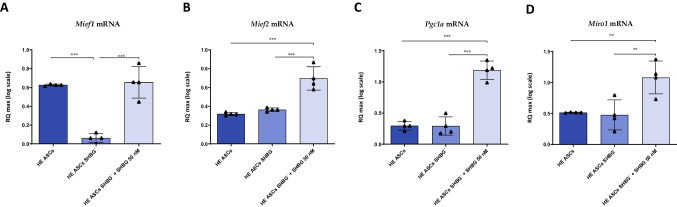
Representative graphs showing the relative expression levels of genes involved in mitochondrial metabolism. The expression of Mief1 (**A**), Mief2 (**B**), Pgc1a (**C**) and Miro1 (**D**) was performed by RT-PCR analysis. Representative data are shown as mean ± SD. **p value < 0.01, ***p value < 0.001

Insofar mitochondrial metabolism is a critical machinery involved in proper ASCs functions, the level of genes expression related to mitochondrial metabolism was analyzed under SHBG-deficiency and SHBG-rescue conditions (Fig. 8). The obtained results showed that the relative expression level of *Mief1* was significantly downregulated in SHBG-silenced cells compared to native cells (Fig. 8A; *p < 0.001*).

Cells treated with exogenous SHBG showed a visible restoration of the same transcript level, which was significantly upregulated when compared to silenced cells (*p < 0.001*) and normalized at a level comparable to that in native cells (Fig. 8A). The relative expression level of *Mief2* was not affected by SHBG knockdown compared to native cells (Fig. 8B). However, it has been found to be upregulated in SHBG-treated cells when compared to silenced cells (*p < 0.001*) and to native cells (*p < 0.001*).

Silencing of SHBG did not influence the expression level of *Pgc1a* compared to native cells (Fig. 8C). This gene was similarly upregulated in exogenous SHBG-treated silenced cells, as compared to silenced cells (*p < 0.001*) and native cells (*p < 0.001*). Analysis of *Miro1* expression haven’t evidenced any visible changes of its expression after SHBG depletion (Fig. 8D), however, treatment of cells with exogenous SHBG resulted in a substantial increase in its transcript level compared to silenced cells (*p < 0.01*) and native cells (*p < 0.01*).

### SHBG Knockdown Negatively Regulates the Basal Adipogenic Potential of EqASCs

**Fig. 9:**
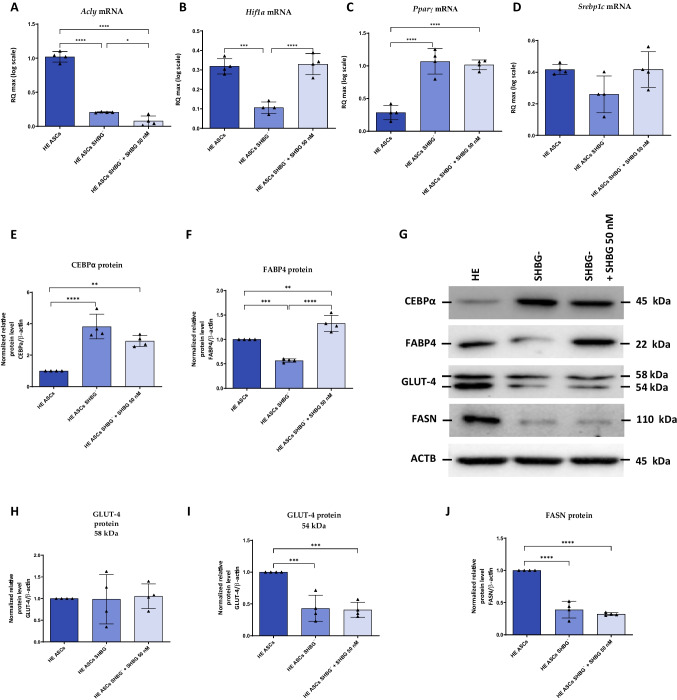
SHBG and adipogenesis regulators interplay analysis. The relative expression levels of *Acly* (**A**), *Hif1a* (**B**), *Pparγ* (**C**) and *Srebp1c* (**D**) were analyzed by RT-PCR. Protein levels of CEBBPα (**E**), FABP4 (**F**), GLUT-4 (**H**, **I**) and FASN (**J**) were analyzed by Western Blot (**G**, representative membranes). Representative data are shown as mean ± SD. **p value < 0.01, ***p value < 0.001 and ****p value < 0.0001

To investigate whether SHBG is involved in the regulation of ASCs’ adipogenesis, the expression of genes involved in the regulation of this process was analyzed at the mRNA and protein levels. The obtained data showed that the relative expression of *Acly* in SHBG-sliced cells was significantly downregulated compared to native cells (Fig. 9A; *p < 0.0001*). Treatment of the cells with exogenous SHBG further reduced this level, which was lower than in silenced cells (Fig. 9A; *p < 0.05*) and native cells (Fig. 9A; *p < 0.0001*). SHBG silencing also resulted in a loss of *Hif1a* expression compared to that in native cells (Fig. 9B; *p < 0.001*). Interestingly, SHBG rescue enabled to restore normal H*if1a* expression was upregulated when compared to silenced cells (Fig. 9B; *p < 0.0001*), and native cells (Fig. 9B).

In contrast, SHBG downregulation significantly increased the relative level of *PPARγ* transcript compared to control cells (Fig. 9C; p < 0.0001). Treatment of silenced cells with exogenous SHBG did not significantly affect the expression level of this gene compared to silenced cells, which was significantly higher than that of native cells (Fig. 9C; *p < 0.0001*).

Further analysis showed that SHBG loss had no significant impact on *Srebp1c* mRNA level, a late adipogenesis marker in both silenced cells and after exogenous SHBG treatment (Fig. 9D). Other key adipogenesis regulators have been further analyzed at the protein level. In SHBG-silenced cells, a marked increased CEBPα protein level was observed compared to native cells (Fig. 9E, G; *p < 0.0001*). In cells treated with exogenous SHBG, the expression of the same protein was reduced however of statistical insignificance compared to silenced cells; moreover, the increased CEBPα protein abundance in SHBG-rescued cells was less significant when compared to native cells (Fig. 9E, G; *p < 0.01*) than in SHBG-depleted cells.

Therewith, SHBG-silenced cells showed critical reduced levels of the anti-adipogenic FABP4 protein when compared to native cells (Fig. 9F, G; *p < 0.001*). After treating the cells with exogenous SHBG, the level of this protein was significantly higher compared to both silenced cells (Fig. 9F, G; *p < 0.0001*) and native cells (Fig. 9F, G; *p < 0.01*) suggesting a FABP4-mediated anti-adipogenic mechanism.

Further analysis showed that silencing of the SHBG protein as well as treatment of the silenced cells with exogenous SHBG did not significantly affect the level of the 58 kDa subunit of the GLUT-4 protein (Fig. 9G, H). However, there was a significant decrease in the level of the 54 kDa subunit of this protein in silenced cells compared to native cells (Fig. 9G, I; *p < 0.001*).

Treatment of cells with SHBG did not affect the level of this subunit, there was no difference from silenced cells, while the amount of protein was still lower than in native cells (Fig. 9G, I; *p < 0.001*). Silenced cells also had a reduced level of FASN protein compared to native cells (Fig. 9G, J; *p < 0.0001*). In this case, treatment of cells silenced with exogenous SHBG also did not restore the level of this protein compared to silenced cells, and its level remained lower than that observed in native cells (Fig. 9G, J; *p < 0.0001*).

## Discussion

Mesenchymal stromal cells (MSCs) represent nowadays a crucial biological tool with high therapeutic value in both human and veterinary medicine. Besides their important involvement in tissues homeostasis maintenance, various lines of evidence suggest that under sustained distress, MSCs may acquire a pathological phenotype, that strongly impairs their biological and therapeutical properties [27]. Hence, the screening for new therapeutic approaches aiming at restoring proper MSCs functions is receiving growing interests. SHBG is a liver glycoprotein involved in the transport and the distribution of various sex hormones, which has been recently pointed out as a critical metabolic regulator, and proposed as a good therapeutic candidate for the management of metabolic disorders including insulin resistance and metabolic syndrome in humans [28]. In the presented study, we investigated the implication of SHBG in the regulation of metabolic activity and basal adipogenic potential of equine adipose-derived stromal cells (ASCs) after SHBG knockdown.

Our obtained results demonstrated that SHBG silencing affected ASCs behavior, evidenced by a change in the cellular metabolic activity and the proliferation capacity. Loss in SHBG protein resulted in a visible modification of initial cellular morphology as well as reduced proliferation and migratory potential. Interestingly, SHBG knockdown decreased the basal level of apoptosis, shown by the lower number of apoptotic cells when compared to native cells. Therewith, treatment of silenced cells with exogenous SHBG did not rescue the cells from impaired early and late apoptosis stages, however increased the expression of nucleic marker of proliferation Ki-67 and the migratory potential of ASCs. Besides SHBG has been previously studied for its effect on various cancer cell lines growth and survival, its influence on non-cancerous cells proliferation and viability has not yet been elucidated. Moreover, SHBG showed differential effects toward various cell types, suggesting distinct mechanisms of action. Recent investigation reported on the importance of SHBG protein in the maintenance of trophoblasts metabolic activity, proliferation and viability. Hence, it has been observed that inhibitory effect of miR-26b-3p toward cell proliferation, invasion, migration, cell cycle and survival correlated with a marked decrease in SHBG protein expression and availability [29]. Otherwise, SHBG has been found to inhibit MCF-7 breast cancer cells growth via the direct blockade of ERK1/2 factors, while it has been demonstrated to stimulate the proliferation of the prostate cancer cell line, ALVA-41throught the increase in intracellular cAMP levels [30]. Cyclic adenosine monophosphate (cAMP) is a crucial second messenger involved in the signal transduction of many cellular processes including growth, apoptosis, migration and differentiation [31]. Intrinsic effects of cAMP strictly depend on the cell target. Chen and al. [32], demonstrated that intermittent parathyroid hormone promotes rate bone mesenchymal stromal cells (BMSCs) proliferation and growth via cAMP/PKA pathway activation. Similarly, Chuang and collaborators [33], found that G protein-coupled estrogen receptor-1 (GPER-1), which shares structural and functional similarities with the SHBG receptor in terms of G protein subunits, that are involved in the conversion of Adenosine Triphosphate (ATP) into cAMP [30, 34], mediates rat bone marrow-derived MSCs proliferation and cell cycle entry through the initiation of the cAMP/PKA/p-CREB axis and upregulation of cyclin D1/cyclin-dependent kinase (CDK) 6 and cyclin E1/CDK2 complex expression and formation. Other studies have also emphasized the implication of cAMP/PKA pathway in the regulation and potentiation of other types of MSCs [35, 36], and highlighted the potential of cAMP activation in enhancing the migration of mouse embryonic stem cell (mESC) through effective remodeling of cell junctions and actin cytoskeleton [37], suggesting that SHBG silencing may impair cAMP/PKA activation and consequently reduce proliferation and migration capacity, which are reversed upon exogenous SHBG supplementation. Sustained growth, proliferation and migration are key elements governing the therapeutic potential of MSCs. Application of MSCs for clinical purposes requires their in vitro expansion, as they naturally occur at low density. Moreover, the efficient mobilization and migration of MSCs from their niche or injection site to the injured tissue is a prerequisite for proper cell-assisted tissue repair et regeneration [38, 39]. Therefore, the presented data indicate that SHBG might possess a good potential in improving the proliferation and mobilization of ASCs and thus could potentiate their pro-regenerative properties.

Apoptosis or programmed cell death is a physiological process that maintains cellular and tissular turnover in coordination with proliferation and division. Being crucially involved in the regulation of tissues remodeling and immune system homeostasis, impaired apoptosis has been previously implicated in the pathogenesis of various dysfunctions including sustained pro-inflammatory response and activation of lymphocyte T cells, as well as endothelial dysfunction and cellular senescence [40, 41]. Moreover, suppressed apoptotic pathway activation has been correlated with reduced therapeutic efficiency of MSCs upon transplantation, in relation to depleted immunosuppressive ability and altered secretome, as well as excessive senescence [42–44]. Our data showcased that SHBG protein is a pivotal regulator of cell proliferation/Apoptosis balance. SHBG silencing induced a significant loss in basal pro-apoptotic processes mediated by the downregulation of the initiator BAX transcript, the reduced number of cells with activated caspase 3/7 axis, and the high level of β-galactosidase-positive senescent cells. Interestingly, SHBG knockdown did not affect the expression of the pro-survival BCL2 gene. Similarly, Wang and colleagues [45], evidenced the anti-apoptotic outcomes of SHBG silencing in the HTR-8/SVneo trophoblast cell line, which has been associated with a concomitant increased ERK pathway activation, further abrogated following SHBG upregulation. Therewith, SHBG protein has been shown to reverse the pro-survival effects of estradiol in the MCF-7 cancer cell line after 48 h incubation [46]; however, in our study, the treatment of SHBG-silenced ASCs with the exogenous protein did not restore the basal apoptosis level; this discrepancy may be explained by the differences in treatment times, as 24 h incubation maybe insufficient for detectable significant changes in the apoptotic tendency of silenced ASCs. Nonetheless, SHBG supplementation induced a marked upregulation of BAX transcript, which was found to be suppressed in the absence of endogenous protein, evoking the initiation of apoptosis rehabilitation within silenced cells in the presence of exogenous SHBG, supported by the observed decreased number of senescent cells following 24 h incubation with exogenous SHBG.

In nonapoptotic cells, BAX depletion has been associated with various pathologic alterations ranging from energy metabolism disruption, reduced calcium disposal to mitochondrial dynamics impairment [47]. In this research, SHBG knockdown engendered a critical failure in mitochondrial dynamics, evidenced by a drop in mitochondrial transmembrane potential, and a disruption in the expression of key dynamics mediators including MFN, PARKIN and PINK at both mRNA and protein levels. SHBG importance in maintaining proper mitochondrial functions has been further confirmed following the addition of exogenous protein to silenced ASCs, which significantly reinstated and potentiated the depleted mitochondrial dynamics and further ameliorated mitochondrial biogenesis as evidenced by the increased number of respiring mitochondria with branching tubules. Correspondingly, megalin receptor-mediated SHBG internalization has been proposed as a part of SHBG intracellular metabolic signaling regulating mitochondrial physiology [48]. In addition, Victor et al. [49], established that a collapsed mitochondrial activity in leukocytes strongly coincided with a critical decrease in SHBG protein levels, suggesting thus the tight crosstalk between mitochondrial health and SHBG activity. Yet, the exact molecular mechanisms underlying SHBG regulatory potential toward mitochondrial function has not been reported. Here we found that altered mitochondrial dynamics correlated with a loss in BAX expression in SHBG-depleted ASCs cells. Indeed, Boohaker and collaborators [50], previously demonstrated the negative impact of BAX deficiency on mitochondrial energetic metabolism. Primary hepatocytes derived from BAX-deficient mice displayed fragmented mitochondrial network, reduced ability to generate sufficient levels of ATP, diminished citrate synthase activity, and excessive glycolysis. Likewise, Bax protein has been reported to regulate mitochondrial morphogenesis and promote normal fusion and elongation, mainly through the activation of GTPase Mfn2 oligomerization and its submitochondrial translocation in the OMM, while its suppression has been associated with mitochondrial tubules shortening and overall decrease in mitochondrial size and network branching [51]. What is more, Bax knockout resulted in a substantial increase in mitochondrial ROS generation in a model of nonapoptotic Neurons [52], which stays in line with our obtained data demonstrating an elevated number of cells with excessive ROS following SHBG silencing that displayed reduced Bax expression, all of which have been reversed after adding exogenous SHBG to cultured cells; hence pointing out the important implication of SHBG in maintaining mitochondrial metabolism and biogenesis, which may at least partly be related to a Bax-dependent mechanism.

Therapeutic potential and clinical significance of MSCs including ASCs lies in their multipotency and ability to undergo trilineage differentiation, which finds its utility in restoring and regenerating injured tissues. The conversion of ASCs into mature adipocytes plays thus a pivotal role in maintaining adipose tissue homeostasis, but also participates in the progression of various metabolic disorders including obesity under unfavorable conditions. Excessive calories intake has thus been correlated to adipose tissue expansion and hyperplasia, mediated by ASCs sustained differentiation [53, 54]. While a wide range of molecular stimuli have been identified in the course of increased adiposity, whether SHBG protein participates in the positive or negative regulation of adipogenesis is still not fully elucidated. Here we found that SHBG depletion led to a visible increased abundance of master pro-adipogenic markers expression, including PPARγ and CEBPα in equine ASCs. Both CCAAT/enhancer binding protein C/EBPα and Peroxisome Proliferator Activated Receptor PPARγ co-operate to positively regulate the complete adipogenic program leading to mature adipocytes generation [55]. During adipose tissue turnover, pro-adipogenic signals elicit the rapid expression of /EBPβ, and C/EBPδ transcription factors that subsequently initiate the transcription of C/EBPα, which in turn activates and maintains the expression of PPARγ, the master regulator of MSCs adipogenic commitment and insulin responsiveness, and collectively induced the sterol regulatory element-binding proteins (SREBPs) that governs the transcription of the genes involved in lipids metabolism and insulate terminal adipocyte differentiation events [54]. Our obtained results suggests thus that SHBG may negatively affect adipogenic potential of ASCs, which correlate with previous findings, in which SHBG has been demonstrated to induce adipocytes dedifferentiation. The same study showed that SHBG protein reduced lipid accumulation in 3 T3-L-derived adipocytes, increased lipolysis and corresponding Glycerol levels, and significantly suppressed the expression levels of CEBPα, PPARγ and SREBP1 [26]. Similarly, Saez-Lopez and colleagues [56], reported that SHBG overexpression protects against high-fat diet-induced white adipose tissue expansion in a model of human SHBG transgenic mice, mainly through lipolysis enhancement and PPARγ downregulation. The exact mechanisms by which SHBG modulates adipogenesis have not been fully described. Although recent study demonstrated that elevated cAMP levels, an-SHBG responsive second messenger and subsequent PKA activation, directly suppresses adipogenic differentiation or 3 T3-L1 preadipocytes in a dose-dependent manner [57], other signaling pathways maybe implicated in the observed effects. Indeed, in our study, we found that SHBG knockdown in horse’ ASCs triggered a critical loss in Fatty acid binding protein 4 (FABP4) and Hypoxia-inducible factor 1-alpha (HIF1-α), two potent anti-adipogenic mediators. Interestingly, the treatment of SHBG-depleted ASCs with exogenous SHBG markedly restored the expression of both markers, and importantly induced overexpression of FABP4 protein in regard to native cells. In adipocytes, FABP4 plays the role of a fatty acids chaperone, which orchestrates the complexation of intracellular lipids to biological targets and their entry to various signaling pathways including energy metabolism. Recently, FABP4 has been shown to exert a critical negative feedback loop, that promotes the proteasomal degradation of PPARγ, thus inhibiting its functions and hampering adipogenesis events [58]. Similarly, HIF1-α signaling, which is mainly involved in the adaptative response elicited under hypoxic microenvironment has been found to specifically dampen PPARγ activity, by targeting its phosphorylation at S112 residue, which further suppresses its transcriptional functions and subsequent adipogenic differentiation program [59]. To date, no clear interaction between SHBG and either FABP4 or HIF1-α has been reported yet, although contradictory effect has been proposed by Yamazaki et al. [26], who suggested that SHBG may downregulate FABP4 gene expression, here we provide for the first time the evidence that SHBG is critical for both FABP4 and HIF1-α expression, and could partly represent a molecular axis governing the anti-adipogenic effect of SHBG, however further investigation is needed to clarify the molecular interplay between SHBG FABP4, HIF1-α and PPARγ.

## Conclusion

The presented findings clearly indicate the mastery role of SHBG in the regulation and maintenance of ASCs proper functions and metabolic homeostasis. SHBG is critical for the preservation of proliferation/apoptosis balance, the adequate mitochondrial biogenesis and dynamics and the limitation of oxidative stress. Moreover, SHBG exerts negative regulatory feedback toward adipogenic differentiation events by promoting FABP4-mediated PPARγ suppression. These observations provide the evidence for the use of SHBG as a potential therapeutic lead for the development of anti-obesity molecular therapy.

## Data Availability

All datasets generated and/or analysed during the current study are presented in the article, the accompanying Source Data or Supplementary Information files, or are available from the corresponding author upon reasonable request.
